# Stretching of *Bombyx mori* Silk Protein in Flow

**DOI:** 10.3390/molecules26061663

**Published:** 2021-03-16

**Authors:** Charley Schaefer, Peter R. Laity, Chris Holland, Tom C. B. McLeish

**Affiliations:** 1 Department of Physics, University of York, Heslington, York YO10 5DD, UK; tom.mcleish@york.ac.uk; 2 Department of Materials Science and Engineering, The University of Sheffield, Sir Robert Hadfield Building, Mappin Street, Sheffield S1 3JD, UK; p.laity@sheffield.ac.uk (P.R.L.); christopher.holland@sheffield.ac.uk (C.H.)

**Keywords:** sticky rouse, tube model, brownian dynamics, silk registration

## Abstract

The flow-induced self-assembly of entangled *Bombyx mori* silk proteins is hypothesised to be aided by the ‘registration’ of aligned protein chains using intermolecularly interacting ‘sticky’ patches. This suggests that upon chain alignment, a hierarchical network forms that collectively stretches and induces nucleation in a precisely controlled way. Through the lens of polymer physics, we argue that if all chains would stretch to a similar extent, a clear correlation length of the stickers in the direction of the flow emerges, which may indeed favour such a registration effect. Through simulations in both extensional flow and shear, we show that there is, on the other hand, a very broad distribution of protein–chain stretch, which suggests the registration of proteins is not directly coupled to the applied strain, but may be a slow statistical process. This qualitative prediction seems to be consistent with the large strains (i.e., at long time scales) required to induce gelation in our rheological measurements under constant shear. We discuss our perspective of how the flow-induced self-assembly of silk may be addressed by new experiments and model development.

## 1. Introduction

**Figure 1:**
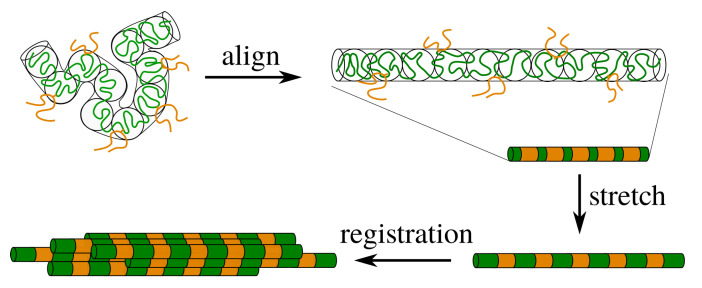
Schematic representation of silk processing. The polymer is entangled and contains stickers (orange) that are connected by linker chains (green). For flow rates that exceed the relaxation rate by reptation, the entanglement blobs of the polymer align in the direction of the flow field. If the chains remain unstretched, the stickers are mobile and the sticker concentration inside the tube is almost homogeneous. Upon modest stretching of the polymer, the distance between the stickers increases and the sticker concentration becomes inhomogeneous, which gives rise to ’registration points’ that enable the correlation of nearby chains.

Protein-based natural materials persistently inspire the development of novel human-made materials, owing to their biocompatibility, unique combinations of strength and toughness [1,2,3,4], low-energy processing [5] and efficient solvent recycling [6]. While the industrial production of polymer-based fibres is challenged by a highly non-trivial interdependence between the molecular level of bond-orientation-dependent nucleation, and the macroscopic level, where the temperature-dependent rheology generates stretch of entire chain segments [7,8,9,10,11], silk is processed in semi-dilute aqueous conditions [5], where nucleation can be induced through the stretch-induced disruption of the solvation layer [12]. In order to generate sufficient stretch at modest flow rates, the silk protein has evolved to contain ‘sticky’ patches (which are assumed to be consisting of ionic calcium bridges between the carboxylated side groups of aspartic and glutamic acids) that significantly slow down stretch relaxation in flow [6,13]. Additionally, it is hypothesised that the sticky patches may serve as registration points for the proteins to correlate their positions and control the nucleation of crystallites [14,15], see Figure 1. This suggests that nucleation takes place shortly after chain alignment. In the present work, we present gelation data under constant shear that reveal that the timescale required for gelation to set is much larger than the time required to align the chains. Using our recently developed tube model for associating polymers [16], we reveal broad distributions in chain stretch that render intermolecular correlation of chains a relatively slow process.

Our starting point is using the insights from several NMR studies [17,18,19,20], and the linear viscoelasticity of silk [21,22] to interpret silk in the gland as a ‘sticky’ intrinsically disordered protein above the overlap concentration [13], akin to entangled associating polymers [23,24,25,26]. The silk protein consists of N=5524 amino acids [22], each with a typical step length of b≈0.4 nm [27,28] (Table 1). In dilute conditions, the radius of gyration, $R_{g}$, is expected to scale as $N^{\nu }$, with ν=1/3 for a poor solvent, 1/2 for a θ solvent and 0.585 for a good solvent. We expect this exponent, which is a measure for the solubility, to depend on the temperature, on the pH and on the cation concentration [13,29,30]. Nevertheless, it was found that the prediction for $R_{g}$ under θ conditions,

(1) $$R_{g}=b\sqrt{\frac{N}{6}}\approx 12.1\;\mathrm{nm}.$$

is in decent agreement with small angle X-ray scattering [22] and small angle neutron scattering [31]. Under these conditions, full chain extension (giving an end-to-end distance of 2210 nm) requires an increase of the end-to-end distance $R_{e}=\sqrt{6}R_{g}\approx 29.7$ nm between the chain ends by a factor of 75.

A polymer stretches in flow when it is unable to sufficiently relax the perturbations imposed by the flow. In dilute conditions, the relaxation is facilitated by long-ranged hydrodynamic interactions with a corresponding Zimm time $\tau_{\mathrm{Zimm}}=\tau_{0}N^{3\nu }$. For a typical monomeric relaxation time of a picosecond, this would imply a Zimm time of just 0.4s, and chain stretching would require enormous flow rates of $2.5\times 10^{6}$ s${}^{-1}$. The first step to improve this to conditions that are biologically achievable by the animal, by concentrating the protein.

Concentrating of the protein has two consequences. First, the chains are overlapping and topological entanglements emerge that restrict to global motion of the chain to curvilinear diffusion within an effective ‘entanglement tube’ with a diameter *a* (which is the statistical length scale below which a substrand appears unentangled and relaxes freely) and a length $aZ_{e}$, with $Z_{e}\approx 10$ the number of topological entanglements per chain [13] (Table 1). The timescale for the protein to escape the tube sets the strain rate required to align a chain. The second consequence of concentrating the chains, which is of importance to stretch an aligned chain (and takes place at higher strain rates), is that the hydrodynamic relaxation mode is done away with, and stretch relaxation is now characterised by the Rouse time, $\tau_{\mathrm{Rouse}}=\tau_{0}N^{2}=\tau_{e}Z_{e}^{2}\approx \times 10^{-5}$ s, with $\tau_{e}\approx 10^{-7}$ s the relaxation time of a strand between entanglements (Table 1).

Although this is two orders of magnitude larger than the stretch-relaxation time in dilute conditions, this is not nearly enough: the animal spins silk at a deformation rate of 1 s${}^{-1}$. The crucial strategy of the silkworm is to incorporate sticky patches in the protein that further slow down the stretch relaxation time to a ‘sticky Rouse’ time [6,23,32], $\tau_{\mathrm{Rouse}}\approx \tau_{s}Z_{s}^{2}$, with $\tau_{s}\approx 10-100$ ms the lifetime of a sticker (which depends on the metal cation composition) and $Z_{s}\approx 5$ the number of stickers per chain [13] (Table 1). In previous work [13,21,22], we have determined the values of the physical parameters, $Z_{e}$, $Z_{s}$, $\tau_{s}$ using the linear viscoelastic response of the silk feedstock. In this work, we will use these values to computationally investigate the stretching of the chains under non-linear flow conditions using our recently developed tube model for association polymers [16].

To model the chain stretching and assess the potential for registration effects, we here extend our tube model with a description for the finite extensibility of the silk protein, as well as a description for the influence of the tension in the stretched chain on the lifetime of the stickers. In the following, we will review earlier work in extension of simple Gaussian chains in relation to the registration effect. Subsequently, we will discuss our methods, including the extension of our tube model and a discussion on the importance to distinguish between an association-dissociation and a bond-swap mechanism for sticker opening and closing under non-linear flow conditions. We then discuss our experimental and simulation results.

## 2. Theory for Registration of Stretched Chains

**Figure 2:**
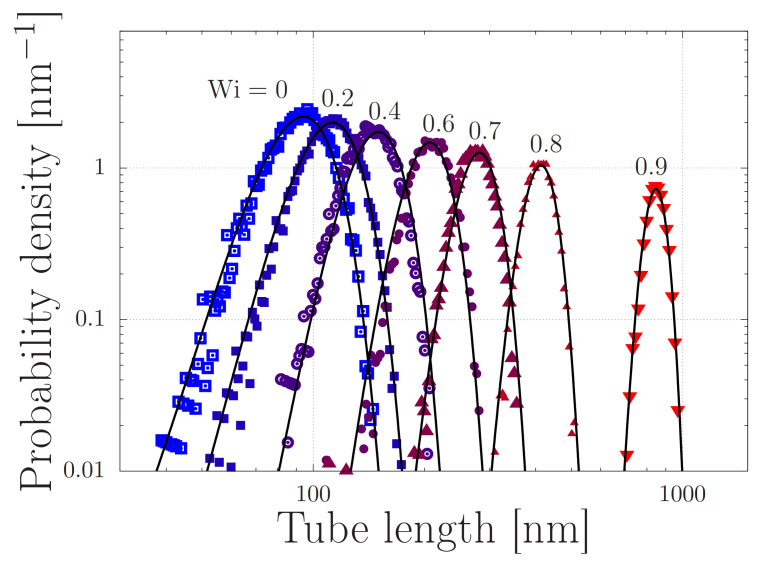
Stretch distributions of regular (non-sticky) polymers with $Z_{e}=10$ at various Weissenberg numbers $\mathrm{Wi}=\dot{\varepsilon }\tau_{R}$ with $\dot{\varepsilon }$ the extensional strain rate and $\tau_{R}$ the Rouse time. The symbols are obtained by simulating Equation (5) in the absence of stickers ($Z_{s}=0$) and without finite chain extensibility ($k_{s,i}=1$). The solid curves are given by Equation (2), with λ=1/(1−Wi).

The registration effect intrinsically relies on the aligned conformation of the polymer chain; in particular, the lengthscale and strength of correlations between the stickers in the flow direction. In order to calculate a correlation function, we will invoke the Gaussian chain approximation (in the next section we will discuss how the presence of stickers changes this idealised, but usually very successful, representation). Our starting point is the equilibrium distribution

(2) $$P\left(R;\langle R\rangle ,\sigma_{R}\right)=\frac{1}{\sqrt{2\pi^{2}\sigma_{R}^{2}}}\exp\left(-\frac{{\left(\langle R\rangle -R\right)}^{2}}{2\sigma_{R}^{2}}\right),$$

that describes the equilibrium statistics of the contour-length fluctuations of a (sub)chain with a mean length 〈R〉 and standard deviation $\sigma_{R}$. We illustrate this distribution in Figure 2, where we consider a chain with $Z_{e}$ entanglements and a tube diameter *a* and no stickers. The tube contour has a mean length $\langle R\rangle =a\lambda Z_{e}$ and a standard deviation $\sigma_{R}=\sqrt{{\langle R\rangle }^{2}/3\lambda Z_{e}}$, where λ is the mean stretch ratio in extensional flow. (We remark that the tube diameter, the number of entanglements and the tube length in principle all depend on the concentration and the solubility of the protein; we assume both properties to be fixed in this work (the tube diameter is $a=bN_{e}^{\nu }\left(\phi \right)$ and the number of entanglements is $Z_{e}=N/N_{e}\left(\phi \right)$, where ν is 1/3 for a poor solvent, 1/2 for a θ solvent and 0.585 for a good solvent. We expect ν to be affected by the temperature, pH and cation concentration [13,29,30]), and $N_{e}\left(\phi \right)\propto \phi^{-1/(3\nu -1)}$ the number of monomers per entanglement strand [33]. Under θ conditions (ν=1/2) the contour length increases linearly with an increasing concentration.).) Hence, the normalised standard deviation is

(3) $$\frac{\sigma_{R}}{\langle R\rangle }=\frac{1}{\sqrt{3\lambda Z_{e}}},$$

which shows that the importance of contour-length fluctuations vanishes with an increasing molecular weight or with an increasing stretch ratio, λ (this is largely the reason of the success of pre-averaged models for the non-linear viscoelastic response of regular (non-sticky) polymers [34]).

**Figure 3:**
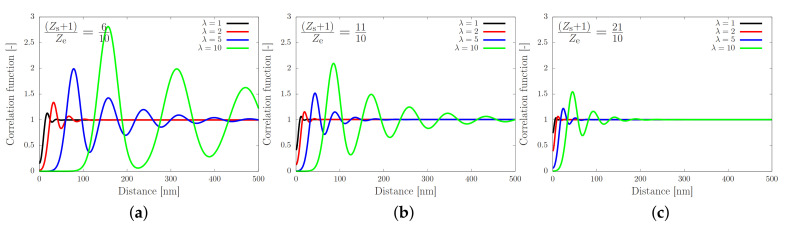
Correlation function against the distance from a sticker as given by Equation (4), for $\left(Z_{s}+1\right)/Z_{e}$ equal to 6/10 (**a**), 11/10 (**b**) and 21/10 (**c**). The curves show an increasing amplitude and an increasing correlation length (distance of the first maximum) for a decreasing number of stickers. The amplitude and length scale are both enhanced by increasing the stretch ratio λ.

We now use this description for a substrand between two neighbouring stickers (n≡1), which represents a portion $f=n/(Z_{s}+1)$ of the chain. The length of this tube segment has a mean $R=af\lambda Z_{e}$ and a standard deviation $\sigma =\sqrt{R^{2}/3f\lambda Z_{e}}$. The next sticker on the chain (n≡2) is separated by a substrand twice this length (in general *n* indicates which sticker in the chain is considered). Ignoring the finite size of the chain, we can define a correlation function as being proportional to the probability of finding a sticker at a distance *r*, as

(4) $$C\left(r\right)\equiv \frac{1}{M}\sum_{n=-\infty }^{\infty }P\left(\lambda ;R_{n},\sigma_{n}\right),$$

with *M* a normalisation factor such that C(r) tends to unity for large *r*, and with $R_{n}\equiv na\lambda Z_{e}/\left(Z_{s}+1\right)$ and $\sigma_{n}\equiv a\sqrt{n\lambda Z_{e}/3(Z_{s}+1)}$. We show the correlation function in Figure 3 for various values of the fraction of stickers and chain extensions.

Figure 3 shows the correlation function as a function of the distance from the sticker. In each panel the stretch ratio is varied, while the only difference between the panels is the number of stickers per entanglement. In panel (a) the number of stickers is 5 per chain of 10 entanglements, corresponding to the silk protein (both values may slightly vary in biological feedstock samples for variations in the concentration and ionic contents [13]). In the absence of stretch (λ=1), the correlation function is rather flat, which implies that there is no long-ranged correlation between the stickers. By modest increases of λ (note that $\lambda_{\max}=23.6$), large oscillations emerge, which point at the correlation of sticker positions in the direction of the flow field. Hence, there is a sensitive rheological switch to create a strongly inhomogeneous sticker concentration in the direction of the flow field. Given that this phenomenon vanishes for an increasing number of stickers per chain (panels (b) and (c)), we speculate that the silk protein has undergone selection to contain slightly fewer than one sticker per entanglement to generate ’registration points’ at modest flow rates, as advertised in the cartoon of Figure 1. However, an important caveat is the fact that we have assumed a relatively narrow Gaussian distribution of chain stretches, while we have recently found that associating polymers have rather broad stretch distribution [16]. We will calculate and discuss the impact of these large fluctuations in the following sections.

## 3. Experimental and Modelling Methods

### 3.1. Experimental Methods

Native silk feedstock (NSF) was obtained from the middle-posterior gland section of commercially bred silkworms, by dissecting 5th instar larvae, as described previously [6,21,22,35]. Generally, a fresh dissection was performed for each rheology experiment and no additional reagents or further chemical treatments were applied to the NSF specimens. Natural variations in the viscosity between different samples can be attributed to variations in ion concentrations (potassium, in particular) that affect the sticker lifetime [6,13]. (These variations in the viscosity can be reproduced by adding ions to diluted fibroin solutions followed by re-concentrating to physiological conditions [36].) A sample of the NSF (ca. 0.1 mL, from the middle-posterior gland section) was carefully transferred to a rheometer (DHR2, TA Instruments) fitted with a CP1/20 geometry (20 mm diameter cone, with $1^{\circ }$ opening angle and 27 m truncation) and a Peltier temperature controlled sample stage. The small amount of excess sample was not trimmed, as the associated stress could initiate gelation. A few droplets of water were placed around (but not touching) the specimen and the sample area was enclosed with a loosely fitting, custom-built cover, to minimise water evaporation from the specimen.

The flow behaviour was measured in several sequentially programmed stages. After a pause of 1 min (to allow thermal equilibration and relaxation of loading flow stress), a constant shear flow rate of $\dot{\gamma }=1$ s${}^{-1}$ at 25 ${}^{\circ }$C was applied over 100 s, to ensure the specimen was uniformly distributed. The low shear rate viscosity ($\eta_{1}$) was obtained by averaging data from the final 30 s. To further reduce the risk of evaporation during subsequent stages of the experiment, the temperature was reduced (at 10 ${}^{\circ }$C/min), while maintaining the shear flow. This also allowed a flow activation energy to be calculated. Next, the linear viscoelastic behaviour was measured by a logarithmic oscillatory frequency sweep (15 steps, ω = 126 to 6.3 rad s${}^{-1}$, at 1% strain followed by 25 steps, 31.4 to 0.13 rad s${}^{-1}$ at 5% strain, with the lower frequency stage repeated to ensure reproducibility). Then, gelation was initiated by applying a faster shear flow (5 s${}^{-1}$); the incipient phase change was observed as a pronounced increase in the shear stress (or viscosity) and first normal stress (i.e., axial force trying to separate the cone from the sample stage). Finally, the progress of gelation was observed using an oscillatory time sweep (at 1 rad s${}^{-1}$ and 5 % strain, over 5 min). In previous work, we identified these structural changes within the sample as the alignment of proteins (consistent with the formation of an aligned, anisotropic gel) through birefringence measurements [5,36], confocal microscopy [37] and rheo-IR [38].

**Figure 4:**
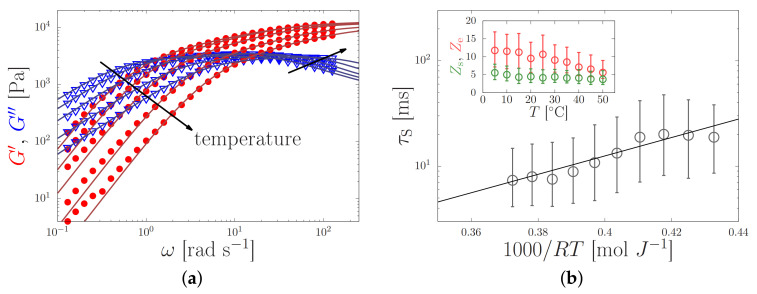
(**a**) Elastic $G^{\prime }\left(\omega \right)$ (dots) and viscous $G^{\prime \prime }\left(\omega \right)$ (triangles) modulus of a silk feedstock with a zero-shear viscosity $\eta_{0}=2554$ Pa s at room temperature against the oscillation frequency, ω, for temperatures in the range of 2–50 ${}^{\circ }$C. The sticky-reptation model (lines) was curve fitted to the experimental data (symbols). (**b**) Temperature-dependent sticker lifetime $\tau_{s}$ (main panel), and the number of stickers $Z_{s}$ (green) and entanglements $Z_{e}$ (red) per chain (inset) as obtained from the fit in the left panel. By curve-fitting the Arrhenius equation to $\tau_{s}$ (solid line) an apparent activation energy $E_{\mathrm{act}}=20.1\pm 14.7$ kJ/mol was found. Both panels are reprinted from Ref. [13] with the permission of the American Chemical Society.

The linear viscoelastic rheology, represented by the elastic modulus $G^{\prime }\left(\omega \right)$ and the viscous modulus $G^{\prime \prime }\left(\omega \right)$, was characterised in two stages. By curve-fitting four Maxwellian modes to the data (Equations (18) and (19)), characteristic time scales and corresponding moduli were extracted [21,35,39]. While this is a convenient method to obtain rapid insight into possible (precursors of) gelation in the sample, the obtained information is phenomenological. For the molecular simulations (see next section), however, we require information about the physical time scales (the reptation time and the sticker lifetime) as well as the molecular (the number of entanglements and stickers per protein). We extract this information from the linear rheology by curve-fitting the sticky-reptation model [13,23,40] to this data, see Figure 4. This latter approach is significantly slower than curve-fitting Maxwellian modes, because both the parameter optimisation and the statistical analysis of the parameter values requires extensive sampling of the parameter space. Ref. [13] extensively discusses this method, and also provides information about the availability of our software.

### 3.2. Tube Model: Brownian Dynamics

Fully atomistic simulations, and even coarse-grained one-bead-per-amino-acid simulations, are computationally unfeasible for the problem at hand. Here, we adopt the central idea by de Gennes and Edwards of replacing the many-chain problem with a single chain in a tube-like confinement imposed by its environment of entanglements [41,42], and solve the Brownian dynamics of the aligned chain in the flow direction (effectively in 1D) [16,43]. This approach is simple yet powerful, and has led to the development of widely applied finite-element solvers [44,45,46,47], a physical explanation for the (apparent) 3.4 power dependence of the relaxation time of polymer melts on the molecular-weight [48], and a comprehensive understanding of the rich non-linear rheology of (bimodal) polymer blends [34,49]. In the spirit of other theory and modelling work on associating polymers [46], we recently added a description for the stochastic attachment and detachment of associating monomers to the tubular environment developed for full non-linear flows [16]. Here, we will add a description for the finite extensibility of the silk chain.

The starting point is to consider a chain consisting of *N* Kuhn segments with length *b*, and $Z_{e}$ entanglements (hence, with tube diameter $a=b{\left(N/Z_{e}\right)}^{1/2}\approx 9.4$ nm). The configuration of the chain is given by the spatial coordinates $R_{i}$ of monomers i=1,⋯,N along the curvilinear direction along the tube, which evolve with time according to the Langevin Equation [34,43,48]

(5) $$\zeta \frac{\partial R_{i}}{\partial t}=\left(F_{\mathrm{thermal},i}+F_{\mathrm{conf},i}\left(R\right)\right)\left(1-p_{i}\right)+\dot{\varepsilon }\zeta R_{i},$$

with ζ is the monomeric friction. The last term in the right-hand side of the equation represents the force exerted by the flow field, with $\dot{\varepsilon }$ the strain rate. The term $F_{\mathrm{thermal},i}$ is a stochastic force given by the equipartition theorem

(6) $$\langle F_{\mathrm{thermal},i}\left(t\right)\rangle =0;\;\;\langle F_{\mathrm{thermal},i}\left(t\right)F_{\mathrm{thermal},i^{\prime }}\left(t^{\prime }\right)\rangle =2k_{B}T\zeta \delta \left(i^{\prime }-i\right)\delta \left(t^{\prime }-t\right),$$

with $k_{B}T$ is the thermal energy, and $F_{\mathrm{conf},i}$ represents the intramolecular forces related to the chain conformation,

(7) $$F_{\mathrm{conf},i}\left(R\right)=\frac{3k_{B}T}{b^{2}}\frac{\partial }{\partial i}\left[k_{s,i}\left(R\right)\frac{\partial R_{i}}{\partial i}\right],$$

which behaves as an harmonic bead-spring model if $k_{s,i}=1$, but using

(8) $$k_{s,i}\left(R\right)=\frac{\left(3\lambda_{\max}^{2}-\lambda_{i}^{2}\left(R\right)\right)/\left(\lambda_{\max}^{2}-\lambda_{i}^{2}\left(R\right)\right)}{\left(3\lambda_{\max}^{2}-1\right)/\left(\lambda_{\max}^{2}-1\right)},$$

includes (approximately [50]) the finite extensibility of the chain up to a maximum stretch ratio $\lambda_{\max}=Nb/Z_{e}a\approx 23.6$ (Nb is the length of the fully extended chain and $Z_{e}a$ is the tube length). The boundary conditions are $\partial R/\partial i=aZ_{e}/N$ both at i=1 and at i=N.

The opening and closing of the stickers are described using the stochastic variable $p_{i}\left(t\right)$, which is zero for open stickers (as well as for for non-sticky monomers) and unity for closed ones. The former type of monomers are unbound and can freely diffuse and respond to the drag exerted by the flow field, as well as relax stress in adjoining segments. A closed sticker, however, is kinetically trapped by its environment and is unable to diffuse or to respond to local stress in the polymer. Consequently, the closed sticker advects with the background flow. In every time step of the simulation, the sticker may close with a constant rate $k_{\mathrm{close}}$ or open with a rate $k_{\mathrm{open}}$ that depends on the local strains in the polymer. (an open sticker closes in a small time step Δt with a probability $1-\exp\left(-k_{i,\mathrm{close}}\Delta t\right)=k_{i,\mathrm{close}}\Delta t+O\left(\Delta t^{2}\right)$ [45]) The underlying physical chemistry of the opening and closing rates is described in the following section.

### 3.3. Physical Chemistry of the Stickers

The rheology of sticky polymers is largely determined by the sticker lifetime. In the case of silk [6,13], the stickers in silk fibroin appear to consist predominantly of calcium bridges between carboxylate-substituted amino acids. This does not preclude contributions from other polyvalent cations, however, such as the smaller amounts of magnesium found in the feedstocks. The lifetime of these ionic bridges depends on their ionic environment and appears to shorten with increasing monovalent cation (mainly potassium) concentration. Usually, the sticker lifetime is described using an empirical Arrhenius form $\tau_{s}=\tau_{s}^{0}\exp\left(E_{\mathrm{act}}/k_{B}T\right)$, where the activation energy is determined from the linear rheology using Van ’t Hoff plots [13,23,26,40,51,52,53]. In this section, we argue that this activation energy might not be the one that is of importance to the non-linear rheology, because a different mechanism of sticker opening takes over.

**Figure 5:**
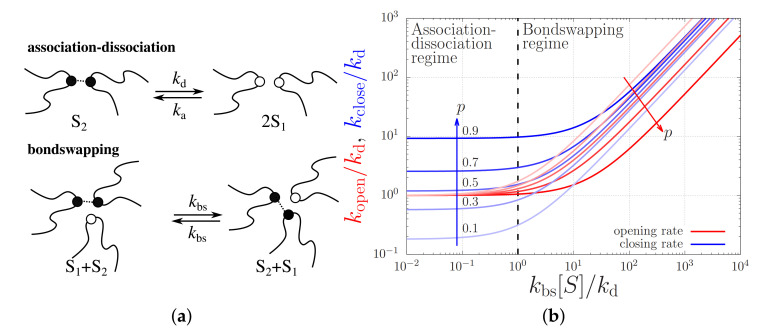
(**a**) Cartoons of the association-dissociation and bondswapping mechanisms. (**b**) Opening and closing rates, $k_{\mathrm{open}}$ and $k_{\mathrm{close}}$, of a sticker against the bondswapping rate $k_{\mathrm{bs}}$. The units are rendered dimensionless using the sticker dissociation rate, $k_{d}$, and the overall sticker concentration, [S]. The curves are calculated for a various fraction of closed stickers, *p*.

Indeed, in most molecular dynamics studies of associating polymers (typically unentangled vitrimers), it is argued that most stickers are bound into closed sticker pairs ‘$S_{2}$’, so that it is unlikely for two free stickers ‘$S_{1}$’ to meet and associate: it is more likely that a free sticker finds a closed sticker pair and closes by bondswapping (Figure 5a illustrates both mechanisms). That is, sticker opening and closing takes place according to the chemical reaction equation

(9) $$S_{1}+S_{2}\overset{k_{\mathrm{bs}}}{\underset{k_{\mathrm{bs}}}{⇌}}S_{2}+S_{1}$$

with $k_{\mathrm{bs}}$ the bondswapping rate. However, under conditions of strong flow the tension in the polymer chains is likely to dissociate the closed sticker pair without the need for a open sticker nearby. Therefore, in the non-linear rheology it is crucial to also include an association-dissociation mechanism,

(10) $$2S_{1}\overset{k_{a}}{\underset{k_{d}}{⇌}}S_{2},$$

with an association rate $k_{a}$ and dissociation rate $k_{d}$.

In order to determine whether or not strong flow alters the mechanism of sticker opening, it is important to first understand which mechanism dominates under quiescent conditions. Hence, we first write down the effective sticker closing and opening rates as

(11) $$k_{\mathrm{close}}=k_{a}\left[S_{1}\right]+k_{\mathrm{bs}}\left[S_{2}\right]$$

and

(12) $$k_{\mathrm{open}}=k_{d}+k_{\mathrm{bs}}\left[S_{1}\right],$$

respectively, where $\left[S_{1}\right]$ is the concentration of open stickers and $\left[S_{2}\right]$ is the concentration of closed sticker pairs. Under quiescent conditions and in chemical equilibrium, these concentrations are $\left[S_{1}\right]=\left(1-p\right)\left[S\right]$ and $\left[S_{2}\right]=p\left[S\right]/2$, with $\left[S\right]=\left[S_{1}\right]+2\left[S_{2}\right]$ the total sticker concentration and *p* the fraction of closed stickers. The fraction of closed stickers is experimentally not known; for now we set p=0.9 (Table 1) in all of our simulations. The value of *p* is controlled entirely by the association and dissociation rate coefficients and by the total sticker concentration through

(13) $$\frac{2p}{{\left(1-p\right)}^{2}}=\frac{k_{a}}{k_{d}}\left[S\right],$$

The effective closing and opening rates, on the other hand, *are* affected by the respective contributions of the bondswapping and the association-dissociation mechanisms, see Figure 5b. This figure plots the opening and closing rates at various fixed *p* values against the total sticker concentration in units of $k_{\mathrm{bs}}/k_{d}$. For small sticker concentrations or, equivalently, small bondswapping rates, i.e., for $k_{\mathrm{bs}}\left[S\right]\ll k_{d}$, sticker opening predominantly takes place by sticker dissociation (i.e., $k_{\mathrm{open}}/k_{d}\approx 1$) and sticker closing takes place by the association to an open sticker, so $k_{\mathrm{close}}/k_{d}\approx 2p/\left(1-p\right)$. In the other limit where the sticker concentration is high, i.e., for $k_{\mathrm{bs}}\left[S\right]\gg k_{d}$, both sticker opening and closing take place predominantly by bondswapping with rates $k_{\mathrm{open}}/k_{d}\propto \left(1-p\right)$ and $k_{\mathrm{close}}/k_{d}\propto \left(p\right)$, respectively.

While in the linear rheology it suffices to know the fraction of closed stickers *p* and the effective sticker lifetime, $\tau_{s}=k_{\mathrm{open}}^{-1}$, in the non-linear rheology the dependence of $k_{\mathrm{open}}$ on the tension within the chain needs to be taken into account. We model this by assuming the chain tension to predominantly affect the dissociation rate

(14) $$k_{d}=\nu \exp\left(-E_{\mathrm{act}}/k_{B}T\right),$$

where ν is an attempt frequency (which may be much smaller than the Rouse frequency of a monomer [13]). In particular, we assume that the force decreases the activation energy for sticker dissociation. In general, the molecular potential is smooth and non-linear; however, to qualitatively capture the force-induced sticker dissociation it suffices to truncate the potential beyond the linear term, so the activation energy may be approximated by [54],

(15) $$E_{\mathrm{act}}=\max\left\{0,\;E_{\mathrm{act}}^{0}-\ell F_{\mathrm{conf}}\right\},$$

with $F_{\mathrm{conf}}$ the conformational force in Equation (7) and *ℓ* nm the typical length scale for bond dissociation (which may depend on the ionic strength [13]). In our simulations, we vary *ℓ* from 0.1 to 100 nm and set $k_{\mathrm{bs}}=0$ and $E_{\mathrm{act}}^{0}=8k_{B}T$ (Table 1). This latter value is obtained from the linear rheology of silk [13], see Figure 4.

For these values, the dissociation of the stickers occurs mainly due the finite extensibility of the chain: A sticker near the chain end experiences a force $F_{\mathrm{conf}}\approx 3k_{B}Tk_{s}\left(\lambda ;\lambda_{\max}\right)\lambda /R_{s}$, and the stretch-dependent sticker lifetime is approximately

(16) $$\tau_{s}=\tau_{s}^{0}\exp\left(-\frac{3\ell }{a}k_{s}\left(\lambda ;\lambda_{\max}\right)\lambda \right).$$

within the harmonic-spring approximation, sticker dissociation is accelerated when the stretch ratio is λ≈a/3ℓ≈89, while sticker dissociation is actually accelerated by the finite extensibility of the chain when λ approaches $\lambda_{\max}=23.6$ due to the divergence of $k_{s}$ (Table 1). Hence, to achieve accelerated sticker dissociation for modest chain stretches the dissociation length *ℓ* should be increased.

### 3.4. Simulation Method

We solve Equation (5) by representing the continuous chain by a chain with a number of ‘beads’ [13,43]: more beads implies a finer discretisation; at rest, the bond length between beads is $dR=a/(N_{\mathrm{beads}}+1)$. The dynamics is simulated by taking fixed-sized explicit Euler time steps. In the absence of stickers and flow, this implies numerical stability for time steps $\Delta t<\frac{1}{2}\tau_{e}/{\left[\pi \left(N_{\mathrm{beads}}+1\right)\right]}^{2}$. In the presence of stickers, every time step should also be smaller than $k_{\mathrm{open}}^{-1}$, which depends on the forces that act on the stickers as discussed above.

## 4. Results

### 4.1. Silk Gelation under Constant Shear

**Figure 6:**
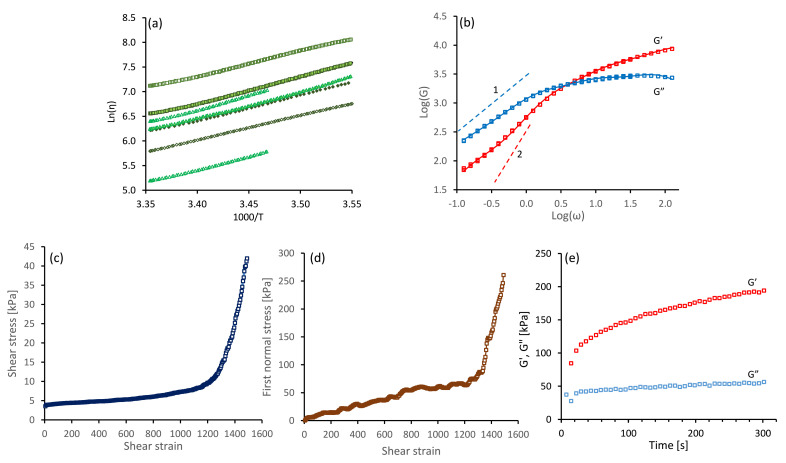
Rheological data measured in shear flow, for specimens of NSF used in this study. (**a**) Arrhenius plots of lnη against 1000/T; different symbols indicate different specimens. (**b**) Exemplar oscillatory data ($G^{\prime }$ and $G^{\prime \prime }$ against the angular frequency, at 15 ${}^{\circ }$C on double logaritmic axes. The continuous red and blue lines indicate the best fit using a Maxwellian model (see Equations (18) and (19) and Table 3). The grey dashed lines indicate the slopes of 1 and 2 expected for $\log G^{\prime \prime }$ and $\log G^{\prime }$ in the ‘terminal’ frequency zone. (**c**,**d**) Shear stress (σ) and first normal stress ($N_{1}$ ) against time at $\dot{\gamma }=5$ s${}^{-1}$ and 15 ${}^{\circ }$C. (**e**) Changes in dynamic moduli (at 1 rad s${}^{-1}$ ) after initiating gelation by flow (as shown in panels (**c**) and (**d**)).

Initial steady shear rate measurements (over 100 s at = 1 s${}^{-1}$ and 25 ${}^{\circ }$C) from all the NSF specimens used in this study gave essentially constant viscosities, indicating good quality solutions without gelation. Values of $\eta_{1}$ ranged from 187 to 2165 Pa s, which encompassed the majority of the stages of fibre spinning during cocoon construction [6]. As noted previously [35], viscosity measurements during the temperature ramps followed Arrhenius-type relationships:

(17) $$\eta \left(T\right)=A\exp\left(\frac{E_{\mathrm{act},\eta }}{RT}\right),$$

where $E_{\mathrm{act},\eta }$ is the flow activation energy and *R* is the gas constant. This is demonstrated in Figure 6a, where plots of lnη(T) against 1000/T gave essentially straight lines, from which it was possible to calculate the flow activation energy $E_{\mathrm{act},\eta }$.

It was found that the offsets of the lines varied considerably, reflecting the differences in viscosities between specimens. Similar variations were found previously [21], and were explained by variations in the concentration of ions in the samples [6,13,36]. Nevertheless, the slopes were similar, giving vales of $E_{\mathrm{act},\eta }$ from 40.6 to 51.6 kJ mol${}^{-1}$. We interpret the activation energy for flow as an apparent one, which emerges due to the temperature-dependence of both the sticker lifetime and the entanglement density. Both properties depend on the composition of the solution (e.g., the concentration of potassium [6,13]). The variations of the flow activation energy for different batches of silk worms (we previously reported 31.8 kJ mol${}^{-1}$ using the same sample preparation technique and rheometry methods [35]) will be subject to future studies.

All the NSF specimens used exhibited viscoelastic behaviour. Exemplar results from an oscillary sweep (at 15 ${}^{\circ }$C) are shown in Figure 6b, for a specimen of NSF with its shear viscosity close to the middle of the range observed ($\eta_{1}=613$ Pa s at 25 ${}^{\circ }$C). Elastic behaviour ($G’>G^{\prime \prime }$) dominated at high frequencies, but changed over to viscous behaviour ($G^{\prime }<G^{\prime \prime }$) at lower frequencies. The dynamic modulus data was reproducible, as demonstrated by measurements from the overlapped high and low frequency steps and the repeated low frequency steps, confirming that the solution was stable.

Following our previous work [13], we extract information about the physical properties of the silk feedstock (elastic modulus $G_{e}$, number of entanglements $Z_{e}$ and stickers per chain $Z_{s}$, and the sticker lifetime $\tau_{s}$) by curve-fitting the sticky-reptation model to the oscillatory results (Ref. [13] provides the model and directions to our curve-fitting software). All extracted physical parameter values are given in Table 2, and lie within the range of values that we obtained from 15 other specimens [13]. We remark that the shortest relaxation time extracted, i.e., the sticker lifetime, is $\tau_{s}\approx 5-31$ ms ($\log\tau_{s}=-1.9\pm 0.4$), while the longest relaxation extracted, the reptation time is $\tau_{\mathrm{rep}}\approx \tau_{s}Z_{s}^{2}Z_{e}/\alpha =0.26$ s, with α=10 a parameter that approximately describes relaxation by contour-length fluctuations [13].

We remark that obtaining a decent fit quality required us to truncate our data for frequencies ω<0.4 rad s${}^{-1}$. Indeed, the oscillatory measurements generally revealed that the NSF specimens were not measured into their terminal zone (i.e., where only the slowest mode contributes to the dynamic moduli), and the expected slopes of 1 and 2 for $\log G^{\prime \prime }$ and $\log G^{\prime }$ in the terminal zone were not observed. In order to quantify these deviations, we also empirically fitted our data using four Maxwellian modes [21,35,39],

(18) $$G^{\prime }\left(\omega \right)=\sum_{i=1}^{4}g_{i}\frac{\omega^{2}\tau_{i}^{2}}{1+\omega^{2}\tau_{i}^{2}},$$

(19) $$G^{\prime \prime }\left(\omega \right)=\sum_{i=1}^{4}g_{i}\frac{\omega \tau_{i}}{1+\omega^{2}\tau_{i}^{2}},$$

where $\tau_{i}$ is a relaxation time constant and $g_{i}$ is a ‘modulus’ contribution’, which combines the actual modulus and the relative abundance of that mode. The values of these parameters are given in Table 3.

**Table 3:** Parameter values obtained by fitting the four-mode Maxwell model in Equations (18) and (19) to the elastic and viscous modulus shown in Figure 6b.

Mode Number, *i*	Modulus Contribution, $g_{i}$ [Pa]	Relaxation Time, $\tau_{i}$ [s]
1	115.5	7.87
2	1935.7	0.531
3	3313.8	0.092
4	5066.0	0.013

We find that the shortest timescale $\tau_{4}=13$ ms lies within the confidence interval (defined by the standard deviation) of the sticker lifetime, $\tau_{s}=5-32$ ms and the modes with time scales $\tau_{3}=0.092$ s $\tau_{2}=0.531$ s seem to capture the shape of the curve for all frequencies near and above the reptation time $\tau_{\mathrm{rep}}\approx 0.26$ s, while the first mode with $\tau_{1}=7.87$ s seems to capture the deviations from the expected slopes of 1 and 2 for $\log G^{\prime \prime }$ and $\log G^{\prime }$ in the terminal zone. Previously [39], we found that the slow-mode contribution builds up throughout pseudo-static shear stress relaxation measurements, and may indicate the formation of precursors to gelation. This slow-mode contribution is relatively small in the samples studied here, and we interpreted the protein to be in a sufficiently good state of solution.

Following these oscillatory shear measurements, we subjected the samples to a steady shear flow at a higher rate. Exemplar data are shown in Figure 6c,d, for the specimen previously characterised in Figure 6b It was found that both shear (σ) and first normal stress ($N_{1}$) increased gradually, up to around 250 s corresponding to a strain of 1250; subsequently both parameters increased more strongly, which was indicative of gel formation. The presence of gelation inferred from the rheometry was corroborated by the observation of enhanced scattering of light from the sample, indicative of inhomogeneities. Finally, gelation was confirmed by observing progressive increases in dynamic moduli (particularly $G^{\prime }$) over time. Clearly, once initiated by flow, gel strength can increase further under essentially quiescent conditions. This also concurs with previously reported observations [39]. The data shown in Figure 6e is typical for a specimen in which gelation has been initiated; however, the elastic modulus remained below the loss modulus, where the shear flow had been stopped prior to the strong increase in shear and normal stress.

### 4.2. Modelling of Dispersed Chain Stretching in Extensional Flow

**Figure 7:**
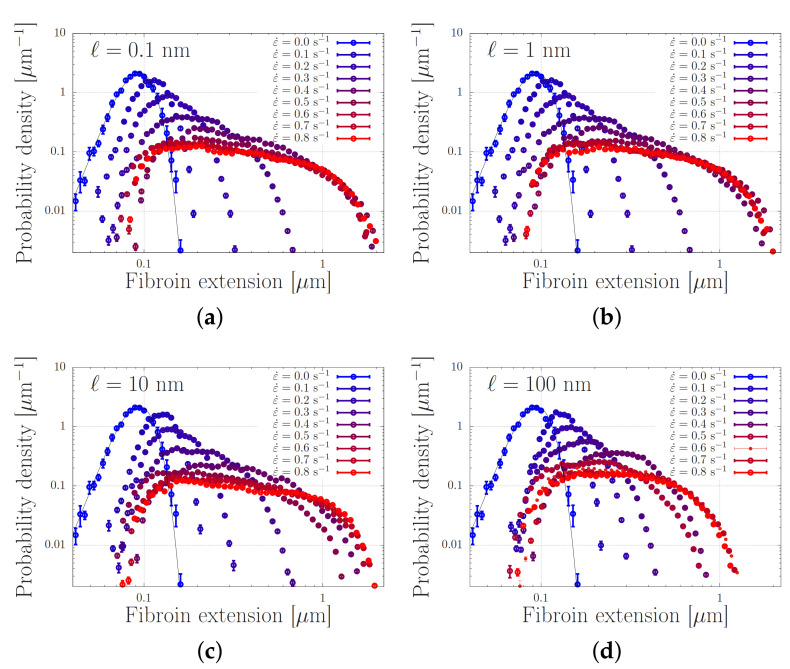
Distribution of chain lengths at various extensional flow rates. The solid curve corresponds to the expected Gaussian distribution when the entanglements are aligned with the flow without further stretching. In the four panels (**a**–**d**), the dissociation length is ℓ=0.1,1,10 and 100 nm, respectively. The parameter values are specified in Table 1.

We present our simulations of Equation (5) with the parameters from Table 1 in Figure 7. In this Figure, every panel shows stretch distributions at various extension rates $\dot{\varepsilon }$, while in each panel the simulations are carried out with a different dissociation length *ℓ* in the range 0.1–100 nm. In the absence of flow, the distributions are Gaussian, as expected from Section 2. Upon increasing the extensional flow rates, the high-stretch tail of the distribution broadens, in agreement with our early predictions of ‘power-law stretching’ in Reference [16]. At high rates, the stretch further increases. For small values of *ℓ*, i.e., for panels (a–c), the stretch distribution is truncated at a stretch of approximately 2 m. In that regime, the finite extensibility of the chain leads to large forces acting upon the stickers and the stickers dissociate. At a value of ℓ=100 nm, the harmonic forces (without finite extensibility effects) are large enough to dissociate the stickers at more modest stretches, and the truncation of the stretch distribution shifts to smaller stretch values.

We emphasise the qualitative difference of these findings compared to what is expected from the usual polymer physics in Figure 2. While the stretch distribution of non-associating polymers narrows down at high flow rates (which suggests the emergence of clearly distinguishable registration points), the stretch distribution of associating polymers widens with an increasing flow rate. At sufficiently high rates, we observe that the stretch-distribution converges to a steady distribution that is independent of the flow rate, provided that the sticky Weissenberg number, $\dot{\varepsilon }\tau_{\mathrm{SR}}$, remains much smaller than the bare Weissenburg number, $\dot{\varepsilon }\tau_{R}$, with $\tau_{\mathrm{SR}}\approx \tau_{s}Z_{s}^{2}$ the sticky Rouse time and $\tau_{R}\approx \tau_{e}Z_{e}^{2}$ the bare Rouse time. This shows that an appropriately designed chain may stretch in flow to an extent that is rather insensivite to the flow rate, while the chemistry of the stickers (which may be altered using cation concentrations and pH and is physically described by the activation energy and dissociation length) seems to be the crucial control parameter.

### 4.3. Toy-Model Simulations of Associating Polymers in Shear Flow

In order to model stresses at small strains due to chain-alignment in extensional flow, and to model stress in shear experiments it will be necessary to model the polymer chains in three spatial dimensions. Nevertheless, with a small adaptation of our 1D model we can already show that the broad distributions of stretch will be preserved, although they will emerge at higher strain rates $\dot{\gamma }$ in shear than the strain rates $\dot{\varepsilon }$ that are needed in extensional flow. The flow field in shear has a *x* and *y* component. We realise that the component in parallel to the flow acts on a length scale comparable to the tube diameter, *a*. Therefore, in Equation (5) we replace the extensional flow field $\dot{\gamma }\zeta R_{i}$ by $\dot{\gamma }\zeta \left(R_{i}-R_{C.M.}\right)$ for $-a/2<R_{i}-R_{C.M.}<a/2$ and $\dot{\gamma }\zeta \left(a/2\right)\mathrm{sign}\left(R_{i}-R_{C.M.}\right)$ otherwise, with $R_{C.M.}$ the center of mass. This implies the extension rate acts only near the center of the chain. Next, in line with our earlier work [16], we consider a sticky dumbbell chain with only two stickers near the ends of the chain, which are synchronised (i.e., either both are open or both are closed; opening and closing of the stickers occurs simultaneously), and we switch off the finite extensibility effects and the effect of stretch on the sticker dissociation time. All remaining parameters are the same as in the previous section, and are summarised in Table 1.

**Figure 8:**
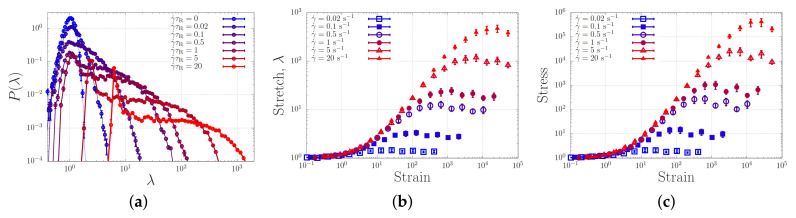
(**a**) Probability density distribution of the chain stretch ratio, λ at various shear rates. (**b**) Stretch as a function of the strain, $\dot{\gamma }t$, at various various shear rates. (**c**) Stress corresponding to the stretch in panel (**b**).

Figure 8a shows the stretch distribution of the dumbbell for strain rates ranging from 0 to $\dot{\gamma }\tau_{R}=20$. In the absence of flow ($\dot{\gamma }=0$), we retrieve a narrow Gaussian distribution. For increasing flow rates, the distribution broadens up and just beyond the ‘sticky’ Weissenberg number $\dot{\gamma }\tau_{R}/\left(1-p\right)=1$ (for a dumbbell, free diffusion is only possible exactly a fraction (1−p) of the time). Beyond the sticky Weissenberg number, we see a broadening of the distribution while a peak emerges at a stretch ratio λ=1, representing the presence of fully relaxed (yet, within our model, aligned) chains. When the bare Weissenberg number of unity ($\dot{\gamma }\tau_{R}=1$) is exceeded, the distribution shifts to higher stretch values. Hence, in analogy with our simulations of associating polymers in extensional flow, we find a broadening of the stretch distribution in shear flow. In contrast to the case of extensional flow, there is, as expected, no runaway stretch.

Filling up the probability distribution of Figure 8a requires finite time. Figure 8a,b show how the average stretch and stress reach the steady state value as a function of the strain $\dot{\gamma }t$, which is the time in units of the strain rate. Panel (c) shows the stress for the same simulations, which approximately equals the square of the stretch, as expected. While at small strain rates the steady state is reached at a strain of the order unity, for larger stretches much larger strains are required. This is in qualitative agreement with the larger strains required to induce gelation in shear experiments (see Figure 6).

## 5. Discussion

In the silk literature, the mechanism of gelation and flow-induced crystallisation of silk is hypothesised to be preceded by the alignment and registration of protein chains. From the view of simple polymer physics, registration points only become discernible when the chains are not merely aligned but also stretched to a small degree. Indeed, silk has approximately 1 sticker per entanglement, and due to the rather large fluctuation of an entangled substrand of the chain, the sticker is able to fluctuate far from its mean-equilibrium position; the sticker concentration is effectively smeared out. By stretching the chain to a small extent in the direction of the flow, the stickers separate to larger distances and a crystal-like correlation function with discernible length scales emerges, see Figure 1.

This view of silk registration is highly idealised, in particular because associating polymers exhibit a broad stretch distribution in steady-state flow. Indeed, akin to ring polymers [55,56], rare events of large chain stretches emerge below the stretch transition. We speculate that the width of the distribution determines how likely it is for equally stretched polymers to associate with each other, such that a correlated ‘registered’ network may form. Recent molecular dynamics simulations of associating polymers in extensional flow do show the influence of flow on the reversible network [57], and indicate that addressing the hypothesis of registration is in reach for molecular simulations.

For such future studies, we suggests to investigate the interplay between chain stretching and registration through the following two possible mechanisms:

1.  The broad stretch distribution may set a probability by which proteins with a similar stretch may ‘register’ or associate and eventually stretch further or give rise to nucleation events; in this case, correctly parameterised single-chain simulations will enable to calculate nucleation rates. Within this mechanism, the finite extensibility of the chains and the tension-dependence of the sticker stability compress the high end of the stretch distribution, and are expected to be important control parameters of the nucleation rate.
2.  At small strains, registration may develop swiftly, so that the stretch of a protein correlates with the stretch of its surrounding proteins, which in turn leads to collective stretching of the entire network.

In order to investigate these hypotheses it will be important gain more insight both experimentally and theoretically into the registration effect, e.g., by mixing in barium (divalent cations) that may bind to the stickers, and performing rheo-SAXS experiments., as well as theoretically using full 3D simulations of the stretching of associating polymers. As we discussed in Section 3.3, currently sticker opening in molecular dynamics simulations is almost exclusively modeled using a bondswapping mechanism; and the activation energy of sticker opening is usually determined from the linear rheology (see Figure 4). Under flow conditions sticker opening will be governed by sticker dissociation, which may have a very different activation energy.

In conclusion, we have applied our recent developments of the established tube model for entangled polymer dynamics, to the more complex case of associating polymers, including the flow-induced stretching of silk. The results indicate that, in spite of the much greater complexity of biomolecules such as the silk protein, over synthetic polymers, that the polymer physics developed over the last 20 years for polymer viscoelasticity can be effective in interpreting the evolved rheological behaviour of silk solutions. It demonstrates, for example, the remarkable effects that well-tailored temporary intermolecular associations can have on the distribution of molecular stretch, in both extension and shear flows. The study also suggests a future direction by which to investigate a potential coupling between the registration effect and broad stretch distributions. We have discussed which new steps, experimental and theoretical, are required to investigate this coupling, itself a promising route to the flow-induced liquid–solid transition that silk must undergo. An understanding of the alignment and stretching of silk at the molecular and microstructural length-scales, such as exemplified in this work, may in future assist the development of novel fibres with similar properties as silk, both in performance and in controlled manufacturing.

## Figures and Tables

**Table 1:** Physical parameter of the silk feedstock within the coarse-grained tube model. Each parameter value is motivated at the point where its symbol is introduced throughout Section 1, Section 2 and Section 3 of the main text. † In our simulations, the bond-swap rate $k_{\mathrm{bs}}$ is set to zero, so the association and dissociation rates, $k_{a}$ and $k_{d}$, are set by the values of *p* and $\tau_{s}^{0}$. ‡  The maximum stretch ratio is defined with respect to the stretch ratio of a chain of which the entanglement blobs are aligned in the flow field (which is therefore pre-stretched to some degree).

Property	Symbol	Value
Number of monomers per chain	*N*	5524
Kuhn length	*b*	0.4 nm
Number of entanglements	$Z_{e}$	10
Number of stickers	$Z_{s}$	5
Entanglement relaxation time	$\tau_{e}$	$10^{-7}$ s
† Quiescent sticker lifetime	$\tau_{s}^{0}$	10 ms
† Quiescent fraction of closed stickers	*p*	0.9
Sticker activation energy	$E_{\mathrm{act}}$	$8k_{B}T$
Sticker dissociation length	*ℓ*	0.1–100 nm
${}^{‡}$ Maximum stretch ratio	$\lambda_{\max}$	23.6

**Table 2:** Physical parameter values obtained by fitting the sticky-reptation model to the elastic and viscous modulus for ω≥0.4 rad s${}^{-1}$ shown in Figure 6b [13].

Physical Property	Value
Elastic modulus, $G_{e}$ [kPa]	8.8±2.8
Number of entanglements, $Z_{e}$	13±7
Number of sticker, $Z_{s}$	4±2
Log sticker lifetime, $\log(\tau_{s}$[s])	−1.9±0.4

## Data Availability

The data presented in this study are available on request from the corresponding author.
